# Infantile Hemangioma of External Auditory Canal

**DOI:** 10.1002/ccr3.5787

**Published:** 2022-04-26

**Authors:** Ali Alsudays, Thamer Albilasi, Mazyad Alenezi, Wala AlShiha, Samir Bawazir

**Affiliations:** ^1^ 37853 Department of Otolaryngology Head and Neck Surgery Prince Sultan Military Medical City Riyadh Kingdom of Saudi Arabia; ^2^ 89660 Department of Otolaryngology Head and Neck Surgery Collage of Medicine Qassim University Qassim Kingdom of Saudi Arabia; ^3^ 37853 Division of Pediatric Otolaryngology Head and Neck Surgery Department of Otolaryngology Head and Neck Surgery Prince Sultan Military Medical City Riyadh Kingdom of Saudi Arabia

**Keywords:** external auditory canal, hemangioma, pediatric

## Abstract

A 14‐month‐old boy who presented with left external auditory canal mass noticed by his parent shortly after birth. Clinically, mass was small, soft and nearly obstructing external auditory meatus. Surgical excision of mass with final histopathological diagnosis confirmed to be hemangioma. Patient followed up for 12 months post‐surgery with no recurrence.

## Graphical abstract

Hemangioma of external auditory canal is rare lesion in pediatric. The hemangioma of EAC should be kept in differential diagnosis of pediatric EAC lesion. The article will assist physician in managing this rare pediatric lesion in proper way.

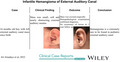

## 1 CASE PRESENTATION

A 14‐month‐old boy presented to our pediatric otolaryngology clinic due to his parent concern of his left external auditory canal mass noticed few months after birth. The mass was slowly increasing in size with no aggravating or relieving factors.

Parent reported history of needle aspiration by primary care physician that yielded a bloody aspirate with reduction of mass size. Shortly after aspiration of the mass, it recurred again. Parent denied history of hearing loss, history of ear discharge, and history of previous ear surgery or any ear trauma.

**FIGURE 1:**
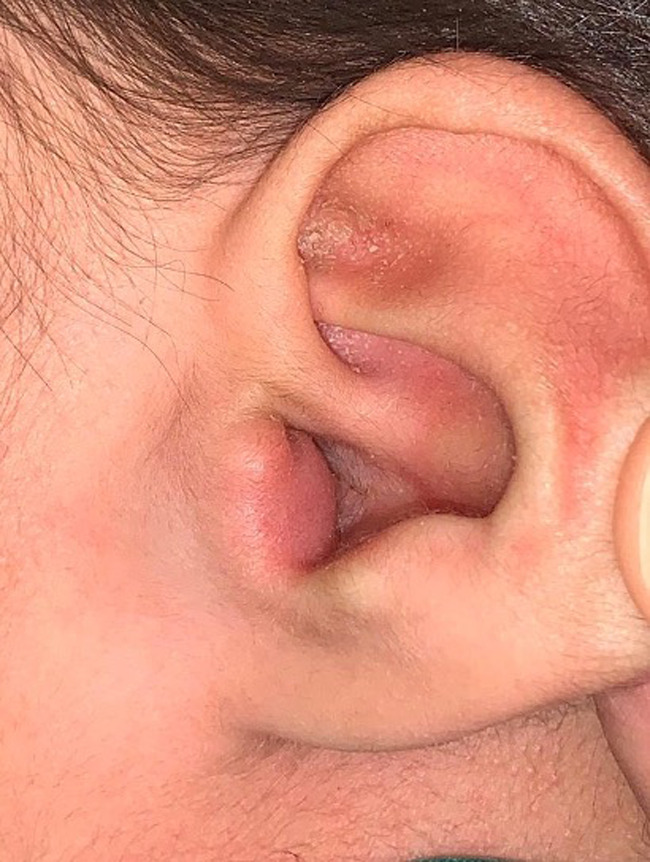
Preoperative finding

Ear examination: Right ear showed clear external auditory canal and an intact tympanic membrane whereas left ear showed a mass that measure 0.5x 0.5 c.m. at external auditory canal meatus. The mass was soft and didn't extend beyond external auditory canal meatus with no skin changes or pits (Figure 1). The mass was nearly occluding the external auditory meatus. The tympanic membrane cannot be seen due to mass occluding meatus with wax impacted behind it. Both auricles were normal.

Preoperative audiological assessment including otoacoustic emission and tympanometry were within normal limits. Due to the difficulty in performing ultrasound (US) with a mass in this area and because of radiation exposure associated with doing a computed tomography (CT scan), no preoperative radiological investigation were done. The presumptive preoperative clinical diagnosis was a sebaceous cyst.

Patient underwent left EAC mass excision under microscope. Intraoperative finding showed that the mass was vascular. Mass was excised completely, and homeostasis was secured with bipolar diathermy smoothly.

The mass was sent for histopathology examination and report came as skin with vascular proliferation consistent with infantile hemangioma. The mass was negative for malignancy.

**FIGURE 2:**
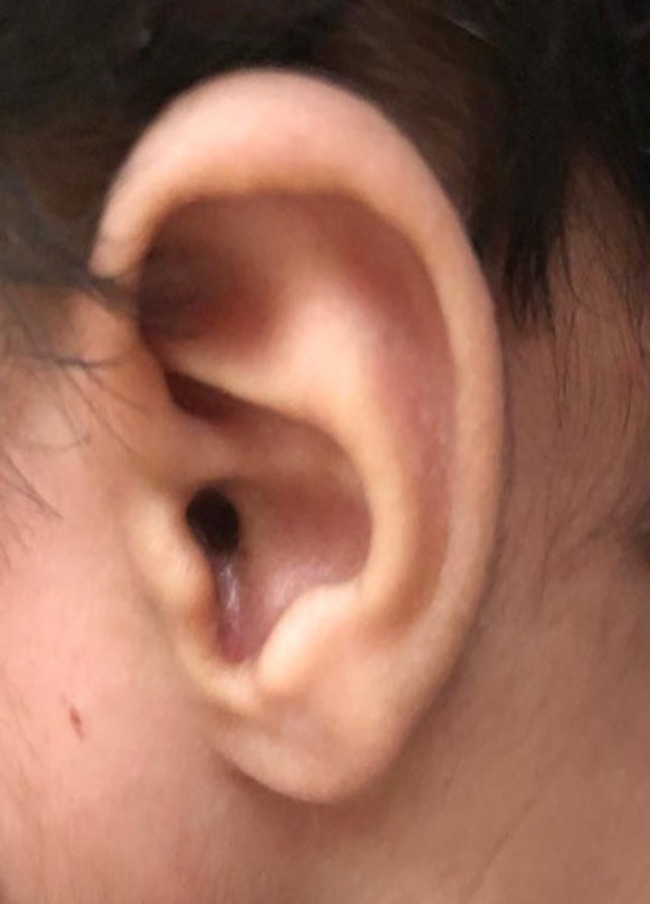
6 months post‐surgery

A follow‐up in the clinic two weeks post‐surgery showed healing surgery area. The patient on 6 months and 1 year post‐surgery follow‐up showed complete healing of the surgical site with no recurrence of the mass (Figure 2).

## 2 DISCUSSION

Hemangioma is a highly vascular benign tumor that commonly found in head and neck.^1^, ^2^ Hemangioma is an extremely rare to be found in external auditory canal. Chen et al. reported that less than 20 adult cases reported to have EAC hemangioma and only 1 pediatric case has been reported.^1^ In terms of histopathology, there are two types of hemangioma the first one is capillary and the other one is cavernous hemangioma.^3^ The most common type of all hemangioma is capillary hemangioma.^2^ Most of the reported hemangiomas in EAC are cavernous. Around 50% of hemangiomas will regress at the age of 5 years.^3^

The presentation of hemangioma varies; some patients will have symptoms others will not. Hearing loss, ear fullness, bloody ear discharge, and pulsatile tinnitus could be the presenting symptoms. Hemangioma in EAC will appear reddish to blue in color, soft with no pulsation or blanching under pneumatic otoscopy.^1^, ^2^

The CT scan of temporal bone is the gold standard in measuring the size, locating the tumor and enables the determination if there is a bone erosion or not.^1^ Angiogram is not essential but can be used to embolize the supplying vessel if bleeding is highly suspected. The confirmatory diagnosis is made after histopathological examination.^1^, ^4^

Surgical excision is the treatment of choice and is indicated if the mass in increasing in size or causing symptoms. Conservative management involves close observation which is preferred for any small and asymptomatic lesion before going for surgical excision.^1^, ^2^ Follow‐ups after the surgery are recommended as there is risk of recurrence.^1^ Zekeriya C reported recurrence of EAC capillary hemangioma recurrence after 7 years of treatment.^3^

The pediatric patient who has been reported by Chen et al. is 12 years old and presented with unilateral hearing loss. On examination, there was a pinkish, soft mass that fully occluded EAC. The hemangioma has been treated surgically by microscopic excision under general anesthesia.^1^

## 3 CONCLUSION

External auditory canal hemangioma is an extremely rare lesion especially in pediatric age group with only a few reported cases worldwide. Throughout history and physical examination with high index of suspicion is crucial.

## CONFLICTS OF INTEREST

The article is self‐fund and no conflict of interest to disclose.

## AUTHOR CONTRIBUTIONS

Ali Alsudays and Mazyad Alenezi involved in data collection and writing of the initial manuscript. Thamer Albilasi involved in data collection. Wala AlShiha and Samir Bawazir involved in revision of the article.

## ETHICAL APPROVAL

Institutional Review Board approval was waived for this report.

## CONSENT

Written informed consent was obtained from the patient to publish this report in accordance with the journal's patient consent policy and available upon request.

## Data Availability

The data are available on request from the authors.
